# Computational Prediction of the Severity of Adverse Drug Reactions Caused by Drug–Drug Interactions

**DOI:** 10.3390/ph19091337

**Published:** 2026-08-24

**Authors:** Vladislav S. Sukhachev, Sergey M. Ivanov, Dmitry A. Filimonov, Anastasia V. Rudik, Vladimir V. Poroikov

**Affiliations:** 1 Department of Bioinformatics, Institute of Biomedical Chemistry, Moscow 119121, Russia; smivanov7@gmail.com (S.M.I.); dmitry.alekseevich.filimonov@gmail.com (D.A.F.); rudik_anastassia@mail.ru (A.V.R.); vvp1951@yandex.ru (V.V.P.); 2 Department of Bioinformatics, Pirogov Russian National Research Medical University, Moscow 117513, Russia

**Keywords:** adverse drug reactions, drug–drug interactions, ADR severity, cross-validation, PASS DDI, PoSMNA, structure–activity relationships

## Abstract

**Background/Objectives**: Adverse drug reactions (ADRs) caused by drug–drug interactions (DDIs) represent an important problem in pharmacotherapy, especially in patients receiving multiple medications. Most computational approaches to DDI-associated ADR prediction formulate the task as a binary classification, but they do not explicitly consider the severity of adverse reactions. Our study aims to develop structure-based models that predict the severity of ADRs associated with specific drug pairs. **Methods**: Datasets were generated using DrugMAP as the source of drug pair–ADR associations with annotated severity categories, and TwoSides was used as an additional source to generate conditionally negative examples. The drug pairs were represented using PoSMNA descriptors, which encode pair-specific structural features derived from the molecular structures of both compounds. Predictive models were built using PASS DDI software. Model performance was evaluated using a modified cross-validation procedure that excluded compound-level overlap between the training and test sets, thereby reducing information leakage caused by the repeated occurrence of the same drugs in different pairs. **Results**: Models were developed for 14 clinically relevant ADR types, including cardiovascular, hepatotoxic, nephrotoxic, hemorrhagic, metabolic, and neurological effects. The unweighted class-level macro-average AUC values ranged from 0.830 for the Major category to 0.911 for the Minor category, while balanced accuracy ranged from 0.776 to 0.857. Predictive performance varied significantly between ADR types and severity categories. Higher accuracy was observed for some ADR types that were better captured by the structure-based descriptors used in this study, whereas complex multifactorial reactions, such as hepatotoxicity, were less accurately predicted. Case-based assessment using clinically documented drug combinations showed that the predicted severity profiles were generally consistent with the expected clinical risk patterns. **Conclusions**: The proposed approach demonstrates that the PoSMNA descriptors of drug pairs can be used for preliminary prediction of DDI-associated ADR severity. The developed models can help to filter out potentially dangerous drug combinations at an early stage and are implemented in the AdverDDIPred web-application.

## 1. Introduction

Adverse drug reactions (ADRs) are among the major challenges in modern pharmacotherapy and have a substantial impact on treatment safety, hospitalization rates, and clinical outcomes [1,2]. Clinical studies and meta-analyses have shown that ADRs are a major cause of complications and impose an additional financial burden on healthcare systems [1].

Current computational approaches to ADR prediction include methods based on chemical structure analysis, machine learning, and the integration of heterogeneous biomedical data [3,4,5,6,7]. However, most existing studies address ADR presence or absence as a binary classification task. This approach limits the practical applicability of such models, because the clinical significance of a reaction depends not only on its occurrence but also on its severity.

Severity-based classification of ADRs as minor, moderate, or major enables more precise assessment of potential risks and may support clinical decision-making. Most existing studies either use binary classification, such as serious versus non-serious ADRs, presence versus absence of an effect, or dangerous versus safe drug combinations, or construct continuous severity scales that are difficult to interpret [8,9,10,11]. Only a limited number of studies have focused on predicting ADR severity [8,11].

Drug–drug interactions (DDIs) are particularly important in the context of ADRs because they substantially increase the risk of ADR development [12]. When several drugs are administered concomitantly, their pharmacokinetic and pharmacodynamic profiles may be altered, which may increase toxicity or reduce therapeutic efficacy. This risk increases with the number of drugs used simultaneously: for example, the probability of ADRs rises from approximately 13% with two drugs to approximately 58% with five drugs and approximately 82% with seven or more drugs [12].

Many ADRs are not detected during clinical trials and are revealed only after a drug has been introduced to the market [13]. Detecting DDI-related ADRs during the concomitant use of multiple drugs is an even more difficult task because of the enormous number of possible drug combinations. Pharmacovigilance systems such as the FDA Adverse Event Reporting System (FAERS) [14] enable the detection of ADR and DDI safety signals by collecting and analyzing spontaneous reports, whereas resources such as TwoSides [13] provide statistically significant associations between drug combinations and ADRs. These data can be used to develop predictive models [13]. Thus, computational prediction methods are becoming increasingly important for identifying potentially dangerous drug combinations before their widespread clinical use.

Data on DDI-associated ADRs are widely available in the literature and in databases such as DrugBank (https://go.drugbank.com/, accessed on 23 June 2026) and Drugs.com (https://www.drugs.com/, accessed on 23 June 2026). Classification models for predicting DDI-associated ADRs have been developed in various studies based on these data and machine learning methods [15]. However, these models do not predict ADR severity. In addition, most studies used human protein target data as features, which limits model development for drugs for which such information is limited or unavailable.

The present study uses an integrated dataset derived from the DrugMAP resource [16,17], which enables analysis of both ADR occurrence and severity classification. In addition to positive drug pair–ADR records with annotated severity categories, conditionally negative examples were generated using a dedicated selection procedure described below in Section 4. Briefly, drug pair–ADR combinations were considered conditionally negative only if they were absent from DrugMAP and were not represented among statistically significant associations with the corresponding ADR in TwoSides after applying additional filtering criteria.

Structure–activity relationship (SAR) models for predicting DDI-associated ADR severity were constructed using PoSMNA descriptors, as described in detail in Section 4. The resulting models were integrated into the AdverDDIPred web application, making them openly accessible to a broad community of researchers and professionals in pharmacology, toxicology, pharmacovigilance, and drug safety assessment. In contrast to many previous approaches, in which representations of drug pairs are constructed from heterogeneous biomedical data, such as target profiles, metabolic enzymes, biological pathways, protein–protein interactions, or previously known drug–drug associations [15], our approach uses only the structural formulas of the two compounds. This allows us to generate a representation of each drug pair directly from the molecular structures using PoSMNA descriptors, without requiring additional biomedical annotations. This is important because such annotations may be incomplete or unavailable for many compounds, whereas structural formulas are usually accessible at earlier stages of drug investigation.

## 2. Results

In this study, datasets were created for 14 ADRs according to DrugMAP terminology: cardiac depression, CNS depressant effect, QT prolongation, hypertension, hypotension, hepatotoxicity, bleeding, nephrotoxicity, bradycardia, hyperglycemia, immunosuppression, hyperkalemia, myelosuppression, and hypokalemia. The available class structure differed across ADR-specific datasets. Moderate and None were represented in all 14 datasets, Major in 12 datasets, and Minor in 2 datasets. The None class was treated as a conditionally negative class and included drug pair–ADR combinations for which no statistically significant association with the corresponding ADR was detected in the analysis of spontaneous reports (see Section 4). SAR models were built using PoSMNA descriptors and PASS DDI software (https://way2drug.com/ddi/ accessed on 23 June 2026).

Table 1 presents the results of SAR modeling for DDI-associated ADR severity classification, including the number of drug pairs in the datasets and predictive-performance metrics. Table 2 shows the average performance of ADRs SAR models.

As shown in Table 1 and Table 2, predictive performance varied across ADR types and severity classes, with class-level macro-average AUC values ranging from 0.830 to 0.911 and balanced accuracy ranging from 0.776 to 0.857.

High metric values were observed for hypotension and hypertension (AUC ≈ 0.90), which may indicate more pronounced SARs for these effects. These ADRs are often associated with well-studied pharmacological mechanisms, such as effects on vascular tone and receptor-mediated regulation, which may facilitate their prediction from chemical structure. In contrast, hepatotoxicity has shown lower accuracy (AUC ≈ 0.76), especially at the moderate severity level. This may be due to the high complexity of hepatotoxicity mechanisms, including metabolic and immune-mediated reactions, which may not be fully captured by SAR analysis based only on chemical structure [18].

Thus, the results indicate that model performance is dependent on the type of ADR. Higher accuracy was observed for ADRs that may be associated with a relatively limited range of pharmacological mechanisms, whereas ADRs involving a more diverse and multifactorial mechanisms, such as hepatotoxicity, were less accurately predicted. The characteristics of the evaluated approaches are summarized in Table 3.

**Figure 1:**
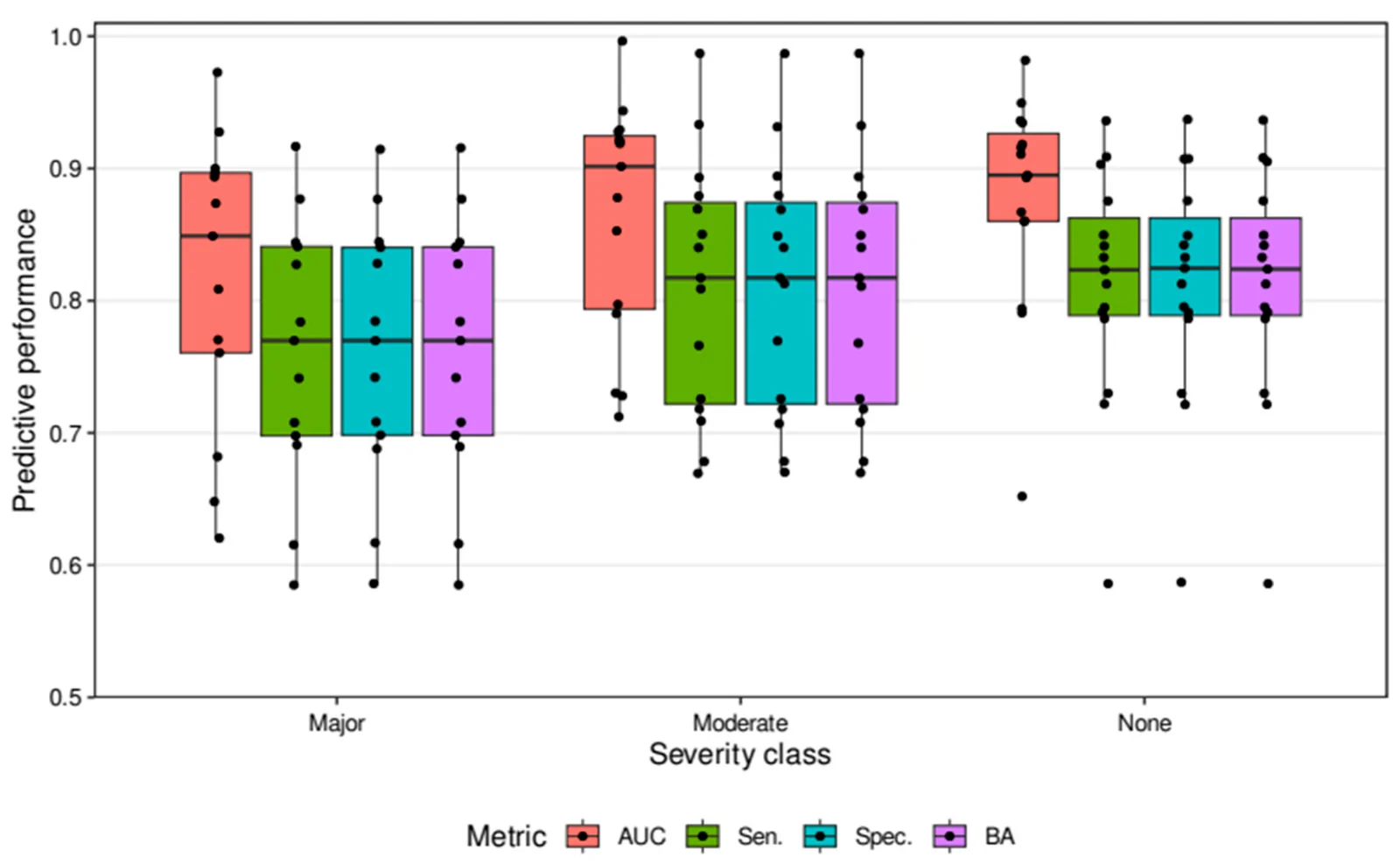
Distribution of predictive-performance metrics across ADR-specific models by severity class. AUC, sensitivity (Sen.), specificity (Spec.), and balanced accuracy (BA) are shown for the Major, Moderate, and None severity categories across the 14 evaluated ADR types. The Minor category is not shown because it was represented in only two ADR-specific datasets.

As shown in Figure 1 and Table 2, the Major class was, on average, predicted with lower accuracy than the Moderate and None classes. This may be related to the smaller sample size for this class: the mean number of drug pairs in the Major class was approximately 27% lower than that in the Moderate class.

To provide a conventional machine learning reference for the proposed approach, a separate benchmark was performed for the bleeding endpoint. The benchmark included a prevalence-based dummy classifier, logistic regression, random forest, and XGBoost trained using Morgan and RDKit molecular fingerprints. Because of the substantial computational cost of evaluating all combinations of algorithms, molecular representations, severity categories, and validation schemes across all 14 ADR-specific datasets, this comparison was restricted to bleeding as a representative endpoint.

The machine learning models were evaluated using both drug-disjoint and conventional pairwise-stratified five-fold cross-validation. Under pair-wise-stratified cross-validation, the same individual drugs could occur in both the training and test sets as components of different drug pairs. By contrast, the drug-disjoint scheme prevented compound-level overlap between the training and test folds.

**Figure 2:**
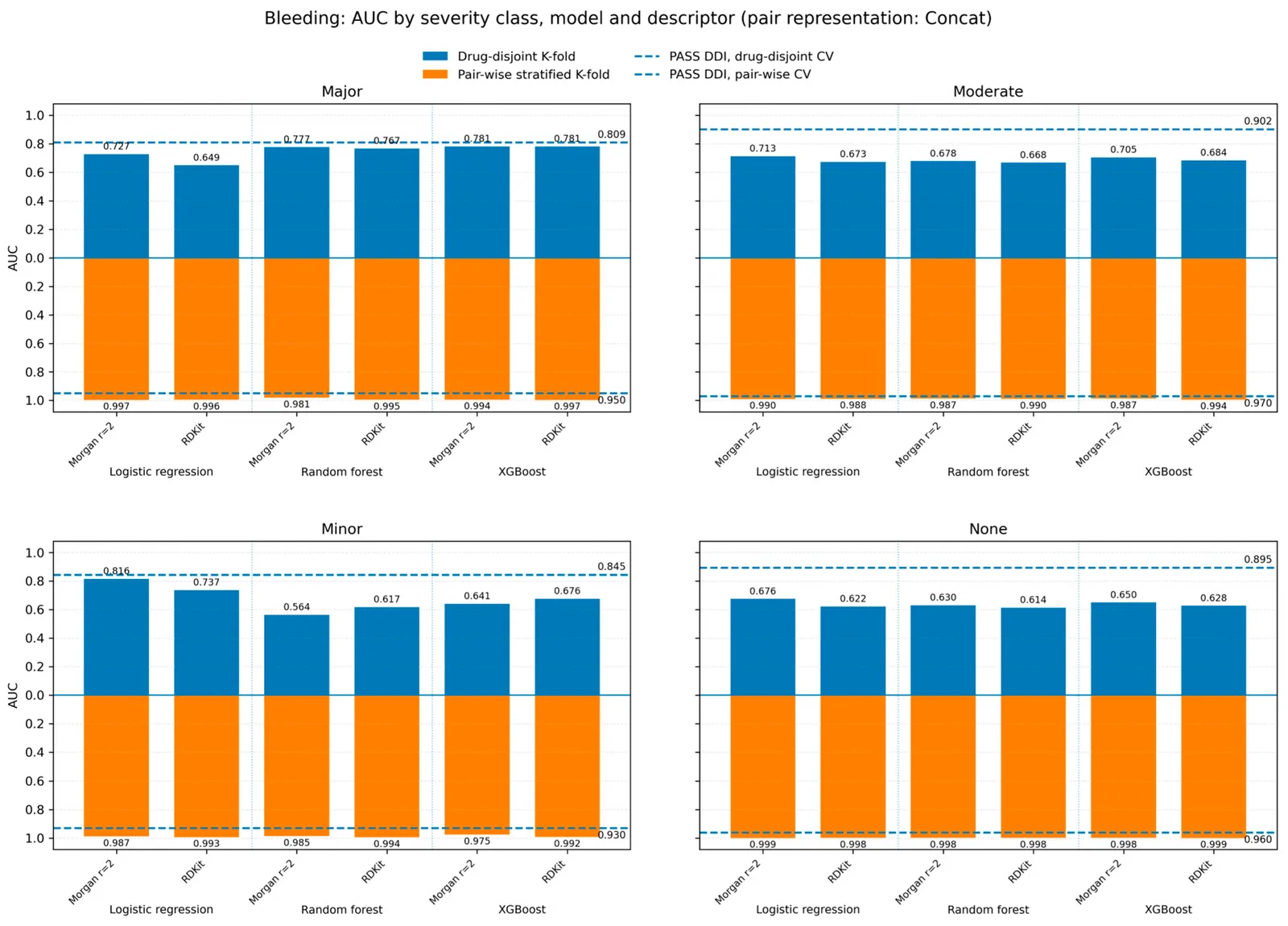
Comparison of ROC AUC values for prediction of bleeding severity using conventional structure-based machine-learning models under drug-disjoint and conventional pair-wise-stratified five-fold cross-validation. Logistic regression, random forest, and XGBoost were evaluated using Morgan and RDKit molecular fingerprints for the Major, Moderate, Minor, and None severity categories. Blue bars above the horizontal zero line represent ROC AUC values obtained using drug-disjoint cross-validation, whereas orange bars below the horizontal zero line represent ROC AUC values obtained using conventional pair-wise-stratified cross-validation; the latter are displayed in the negative direction only for visual comparison, while the corresponding ROC AUC values are positive. The upper blue dashed horizontal line indicates the corresponding PASS DDI ROC AUC obtained using drug-disjoint cross-validation, whereas the lower blue dashed horizontal line indicates the PASS DDI ROC AUC obtained using conventional pair-wise-stratified cross-validation. The blue solid horizontal line at zero serves as a reference line separating the two mirrored sets of bars. Under pair-wise-stratified cross-validation, individual compounds may occur in both training and test folds as components of different drug pairs, whereas drug-disjoint cross-validation prevents such compound-level overlap.

The validation strategy had a pronounced effect on the estimated predictive performance. For the Major bleeding category, conventional pairwise-stratified cross-validation yielded ROC AUC values ranging from 0.981 to 0.997 across the evaluated classifiers and fingerprint representations. Under drug-disjoint cross-validation, the corresponding ROC AUC values ranged from 0.649 to 0.781. Thus, conventional pairwise splitting produced substantially more optimistic estimates than compound-level separation. The same comparison was performed for all four bleeding severity categories (Major, Moderate, Minor, and None), as summarized in Figure 2. Across the evaluated configurations, the magnitude of the difference between validation schemes varied between severity categories and classifiers, but pairwise-stratified cross-validation generally produced more optimistic performance estimates than drug-disjoint validation. This observation demonstrates that compound-level overlap can substantially affect apparent model performance in drug-pair prediction tasks.

To demonstrate the applicability of the developed models, we also performed case-based assessment using specific drug pairs that are widely used in clinical practice. The results are shown in Table 4.

The case-based assessment included examples of predictions that were consistent with published clinical evidence as well as examples illustrating limitations of the model. The individual cases are discussed below. For the combination of warfarin and amiodarone, the Major class was not predicted (*Pa*/*Pi* = 0.090/0.691) and should therefore be considered a false-negative prediction for the Major severity category. At the same time, the model predicted both the Minor (*Pa*/*Pi* = 0.803/0.075) and Moderate (*Pa*/*Pi* = 0.767/0.023) categories. Thus, although the model identified an association of this drug pair with bleeding, it underestimated its severity [19]. This example illustrates an important limitation of the structure-based approach: clinically significant severity may depend on pharmacokinetic interactions, dose, monitoring, and patient-specific factors that are not represented by molecular structure alone. Accordingly, the case-based assessment should be regarded as illustrative rather than as an independent validation of the model.

In contrast, for the combination of warfarin and aspirin, the highest *Pa* value was obtained for the Major class (*Pa*/*Pi* = 0.942/0.035), which is consistent with data showing an increased risk of serious bleeding with concomitant use of anticoagulants and antiplatelet agents [20]. Thus, the model distinguishes between two drug combinations associated with bleeding but characterized by different levels of clinical risk.

For azithromycin and diltiazem, the model predicted the Moderate category (*Pa*/*Pi* = 0.665/0.072), whereas the published case described profound QTc prolongation with cardiac arrest [21]. Thus, the model identified the QT-prolongation association but may have underestimated the severity of the reported clinical outcome. By contrast, for the combination of quetiapine and escitalopram, the model predicted a high risk for the Major class (*Pa*/*Pi* = 0.932/0.007), which is consistent with a reported clinical case of pronounced QT-interval prolongation [22].

For the combination of tramadol and venlafaxine, which is associated with serotonin syndrome and CNS toxicity, the model also assigned the highest *Pa* value to the Moderate class (*Pa*/*Pi* = 0.501/0.119) [23]. This result is consistent with the clinical nature of serotonin syndrome, the severity of which can range from moderate manifestations to life-threatening conditions depending on dose, duration of administration, and individual sensitivity.

The result for the control combination of levothyroxine and amlodipine should be noted separately. This combination is widely used in geriatric practice [24]. No data on clinically significant interaction or contraindications to co-administration were found in the literature or in drug-interaction reference resources, including Drugs.com. At the same time, the model assigned this combination a high probability of belonging to the conditionally negative None class (*Pa*/*Pi* = 0.876/0.012). This indicates the ability of the model not only to identify potentially dangerous drug pairs but also to distinguish them from combinations without a recorded clinically significant risk.

Overall, the case-based assessment results show that the models reproduce the expected risk profiles for several clinically documented drug combinations. At the same time, these data should be interpreted with caution: ADR severity described in a single clinical case is not always equivalent to the generalized severity of a drug interaction, because the clinical outcome depends on dosage, concomitant diseases, liver and kidney function, patient age, and other factors not included in the structural model.

In several cases, the Major and None classes demonstrated higher metric values than the Moderate class. This may be because the Moderate class represents an intermediate severity category and is more likely to include clinically heterogeneous cases, whereas the Major and None classes may have more clearly defined boundaries.

**Figure 3:**
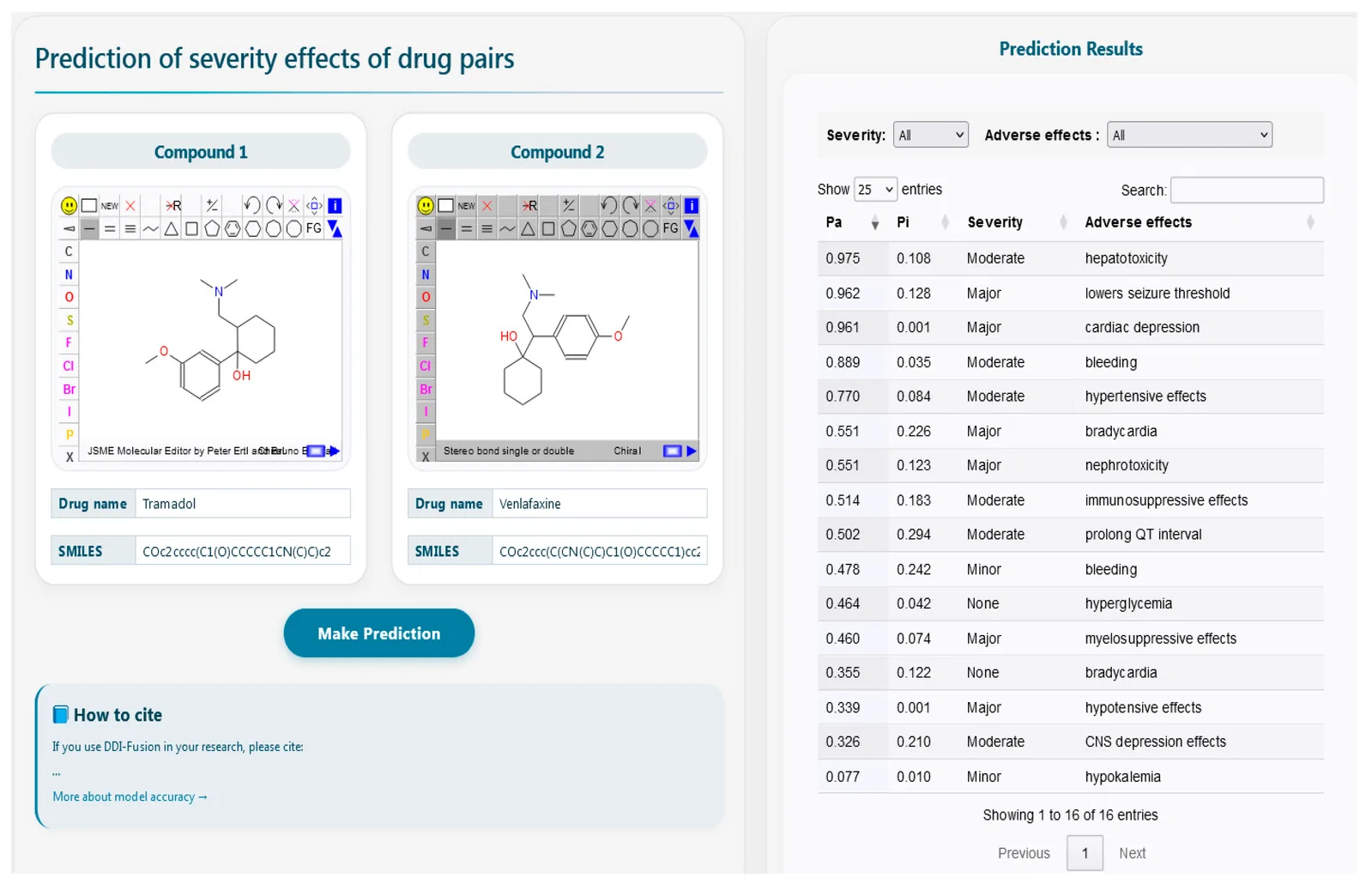
Web interface of the AdverDDIPred application implemented on the Way2Drug platform. Users can submit a drug pair using compound names, SMILES strings, or molecular structures and obtain *Pa* and *Pi* estimates for DDI-associated ADR severity categories satisfying the predefined PASS criterion *Pa* > *Pi*.

All 14 developed models are freely available as part of the Way2Drug resource at AdverDDIPred web application (https://way2drug.com/AdverDDIPred, accessed on 23 June 2026). To obtain a prediction, the user can enter the structural formulas of two compounds by specifying their names, entering SMILES strings, or drawing the structures in the JSME applet [25]. The web application returns *Pa* and *Pi* values for ADRs and severity categories for which *Pa* > *Pi* (Figure 3). *Pa* and *Pi* are independently calculated probability estimates of the presence and absence of a given activity, respectively; in the present context, they correspond to the probability that a drug pair will or will not be associated with a specific ADR at a specific severity level. The *Pa* > *Pi* threshold is applied uniformly to all ADR types and severity categories available on the website.

## 3. Discussion

This study addresses the classification of DDI-associated ADRs according to severity based only on the structural formulas of drugs. The analyzed objects are drug pair–ADR associations for which the severity category must be determined: Major, Moderate, Minor, or None. The task was formulated as a prediction of the severity-specific DDI-associated ADRs for drug pairs. The Major, Moderate, and Minor classes were treated as severity levels, whereas the None class was treated as a conditionally negative class. This approach may be useful for drugs with limited experimental data, because the model input does not require information on known targets, metabolic enzymes, biological pathways, protein interactions, or previously reported DDI; instead, the prediction can be generated directly from the structural formulas of the two compounds alone. In contrast to more common formulations of predictive tasks, such as ADR presence/absence or severe/non-severe classification, the proposed approach enables differences in ADR severity and their potential impact on treatment safety to be considered in greater detail.

Previous studies have addressed related aspects of ADR and DDI prediction using different task formulations. Thakkar et al. developed the DILIst classification for drug-induced liver injury severity at the level of individual drugs [8], whereas Lavertu et al. investigated quantitative assessment of ADR severity using information derived from social media [5]. Other computational approaches, such as the graph-based model of Zitnik et al., focused on predicting polypharmacy-associated side effects of drug combinations rather than explicitly assigning ADR-specific severity categories [15]. In contrast, the present study formulates the problem as severity prediction for a specific ADR associated with a specific pair of drugs and distinguishes Major, Moderate, Minor, and conditionally negative None categories. Thus, the proposed approach complements previous work by addressing the intersection of drug-pair-specific ADR prediction and severity classification using only structural information.

This task has several specific features. First, class imbalance may occur, with severe reactions represented by fewer examples than moderate and mild reactions. In the present study, this is reflected in the lower average predictive performance for the Major class: an average AUC value of 0.809 compared to 0.864 and 0.911 for the Moderate and Minor classes. Second, the boundaries between severity categories may be unclear due to annotation uncertainty and differences between data sources. Finally, because of the specific nature of the subject area, strictly confirmed negative examples are unavailable, which introduces additional uncertainty into the training dataset.

Considering these factors, prediction of ADR severity is a nontrivial problem that requires careful selection of modeling methods and cautious interpretation of the results.

It should be noted that differences in predictive performance between severity categories cannot be explained by sample size alone. According to Table 2, the Minor class has the lowest average number of positive examples (N = 87), but it is characterized by the highest mean AUC (0.911), whereas the None class is represented by the largest number of examples, but its AUC is intermediate. Therefore, the lower mean predictive accuracy for the Major class probably reflects not only quantitative differences between datasets but also annotation characteristics, heterogeneity of clinical ADR manifestations, and differences in the mechanisms underlying individual effects. Another limitation is the use of conditionally negative examples: the absence of a recorded association between a drug pair and an ADR does not guarantee the actual absence of that effect. This may lead to hidden positive examples being assigned to the negative class and introduces uncertainty into the evaluation of model performance. Therefore, the obtained metrics should be interpreted in light of the limitations of the source data and the adopted dataset formation scheme.

The obtained results show that the proposed approach can be used to predict the severity of DDI-associated ADRs; however, predictive accuracy depends substantially on the type of effect considered and on the quality of the source data. AUC values varied widely, approximately from 0.620 (Bradycardia, Major) to 0.996 (Cardiac depression, Moderate) depending on the ADR category and severity class, indicating that task complexity differs across ADR types. Notably, a complex ADR type such as hepatotoxicity was characterized by lower predictive accuracy even when larger training datasets were available. The variable performance observed for hepatotoxicity may reflect the heterogeneous mechanistic basis of drug-induced liver injury (DILI). Structure-based descriptors are expected to be more informative when hepatotoxicity is associated with intrinsic chemical liabilities, such as structural alerts, formation of reactive metabolites, or direct membrane and mitochondrial damage. However, DILI is also strongly influenced by biological context, including hepatic metabolism, transporter-mediated exposure, immune susceptibility, and dose-dependent bioactivation. These factors are not represented explicitly by molecular structure alone. Consequently, structural descriptors may capture only part of the mechanistic basis of DILI, which may explain why predictive performance for hepatotoxicity remained relatively modest despite the comparatively large number of training examples.

An additional limitation is the use of conditionally negative examples. Negative examples were generated based on the absence of a recorded or statistically significant association between a drug pair and a specific ADR in the data sources used (see Section 4). This approach does not guarantee the actual absence of a clinically significant DDI, because some potential ADRs may be unreported, insufficiently studied, or below the threshold of statistical significance. As a result, false-negative examples may be included in the negative (“None”) class, thereby affecting model accuracy. In particular, performance values may be overestimated when easily separable negative examples predominate and underestimated when mislabeled examples are present in the negative class. Nevertheless, this approach is practically necessary because pharmacovigilance systems and clinical literature primarily document the presence of ADRs, whereas the confirmed absence of a specific ADR for a given drug pair is generally not recorded.

In addition to limitations of the source data, this study also has methodological limitations because it does not account for several factors that are critically important for ADR development, including pharmacokinetics and metabolism, dose dependence, individual patient characteristics, and the context of concomitant drug use. This is especially important for complex reactions such as hepatotoxicity, where metabolic pathways and individual sensitivity play a key role. Nevertheless, this limitation is common to most SAR modeling studies, because the data required to incorporate such factors are even scarcer than data on ADR severity.

The developed models have been implemented in the AdverDDIPred web application, which is freely available through the Way2Drug platform at https://way2drug.com/AdverDDIPred. This resource enables users to obtain predictions of DDI-associated ADR severity directly from the structural formulas of two compounds. Users can submit a drug pair by entering compound names, SMILES strings, or drawing structures in the embedded molecular editor. The web application returns *Pa* and *Pi* values for ADRs and severity categories for which *Pa* exceeds *Pi*, thereby providing a preliminary structure-based assessment of potentially clinically relevant DDI-associated ADRs.

## 4. Materials and Methods

The DrugMAP resource (https://drugmap.idrblab.net/, accessed on 23 June 2026) was used as the main data source for DDI-associated ADRs [16,17]. DrugMAP is a database established in 2022 and updated in 2024 that contains information on drug compounds, their targets, DDIs, and pharmacological effects. The database includes data on tens of thousands of compounds and hundreds of thousands of interactions, including drug-pair-associated ADRs and their severity categories: major, moderate, and minor.

The ADR severity categories for drug pairs were manually annotated by the DrugMAP authors using information from the FDA Orange Book, ClinicalTrials.gov, official reports from pharmaceutical companies, and literature data [16,17]. In addition, bibliographic databases were searched to refine and expand the dataset.

The TwoSides database [13], constructed from spontaneous reports in FAERS, was used as an additional data source for DDI-associated ADRs [13]. This resource is intended for studying ADRs that occur during concomitant drug use [13].

TwoSides contains drug pair–ADR associations identified using statistical signal detection methods applied to spontaneous reports. In this study, TwoSides was used to generate negative examples (None class), that is, drug pairs for which no association with a given ADR was recorded. Data derived from the analysis of spontaneous reports, including data from TwoSides, are characterized by substantial noise and incompleteness [26]. Nevertheless, a similar approach has been used successfully in previous work [27].

The training dataset was generated by integrating data from DrugMAP and TwoSides.

Positive examples were drug pair–ADR records extracted from DrugMAP [16,17] for which a severity category (major, moderate, or minor) was explicitly specified. Each drug pair was assigned a class label, e.g., “major, hepatotoxicity” or “moderate hepatotoxicity”.

During data preparation, cleaning and normalization were performed, including duplicate removal, standardization of drug names, and harmonization of ADR terminology. Structural normalization of compounds included standard preprocessing procedures such as salt and solvent removal, charge and protonation-state normalization, and dearomatization/Kekulization. If several records were available for the same drug pair and ADR, the highest severity class among these records was assigned to avoid contradictions in labeling. The conversion of input structures into a standardized internal representation is automatically performed by PASS DDI during the descriptor generation process.

Negative examples were generated from TwoSides [13] as drug pair–ADR combinations that were absent from DrugMAP and were not represented among statistically significant associations with the corresponding ADR in TwoSides [13]. To reduce possible errors in defining negative examples, additional processing was performed, including selection of only those associations that satisfied the specified thresholds for the number of reports and statistical error. The algorithm is described in more detail in our previous work [27].

Despite the strict criteria used to generate negative examples, these examples cannot be confirmed as true negatives and were therefore considered conditionally negative. This means that some of them may potentially correspond to real ADRs that have not yet been reported or have not been sufficiently studied.

To describe the chemical structures of drug pairs, this study used PoSMNA descriptors, which are based on Multilevel Neighborhoods of Atoms (MNA) descriptors and encode molecules as formalized features that are suitable for subsequent machine learning.

MNA descriptors represent a molecule as a set of strings describing atomically centered fragments of the molecule [28]. For each atom, its neighborhoods of different levels are generated sequentially, including neighboring atoms and types of bonds. Thus, a molecule is encoded as a set of strings, that is, linear notations of fragments reflecting its topological structure. This approach effectively captures local structural features that often play a key role in determining biological activity and toxicity [28].

**Figure 4:**
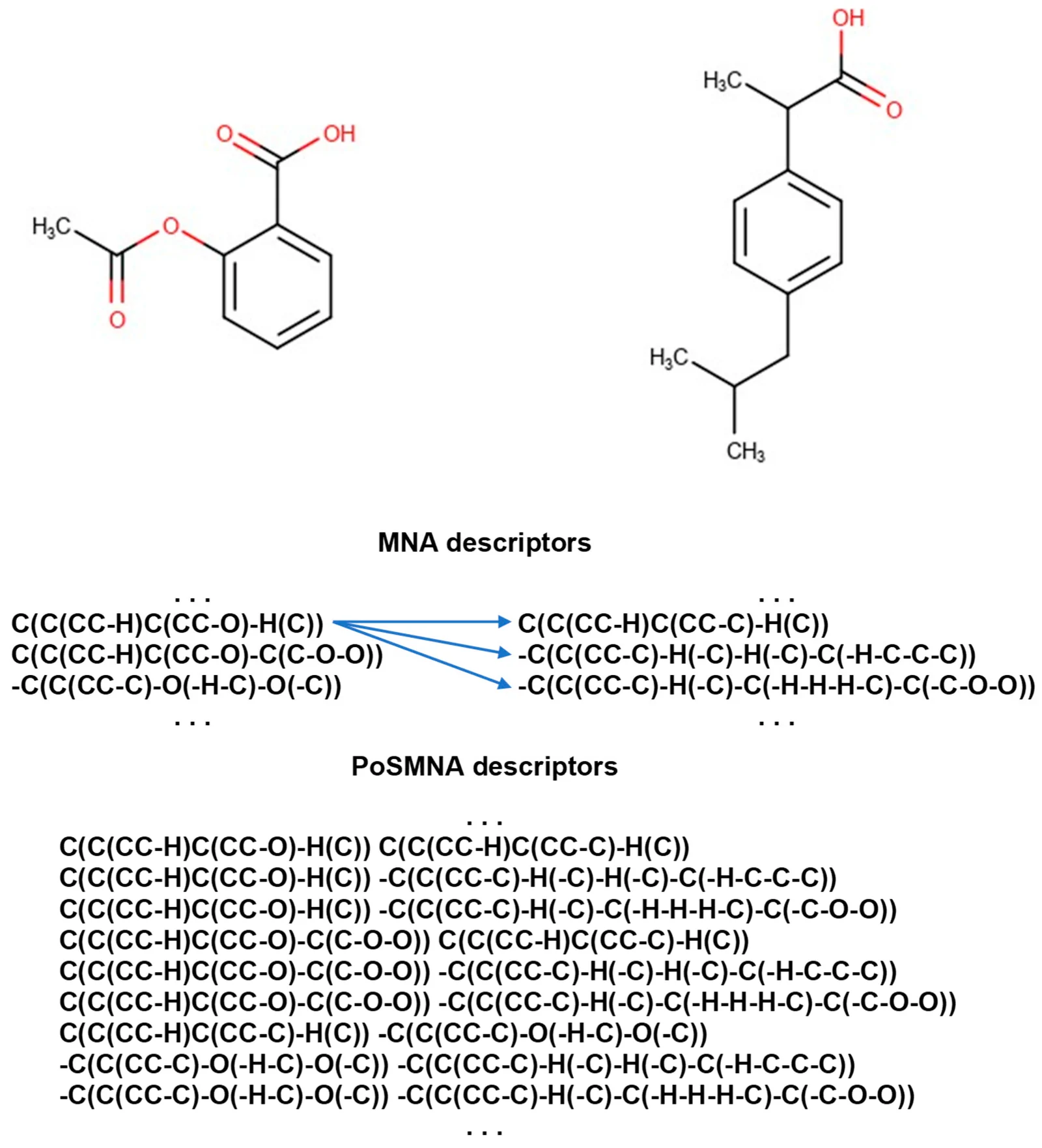
Illustration of the PoSMNA generation algorithm from MNA descriptors. Each MNA descriptor of one molecule is paired with each MNA descriptor of the other molecule. The set of unique combinations constitutes the molecular representation of a drug pair by the PoSMNA method. The structures were chosen arbitrarily, and the images were taken from PubChem [31] for illustrative purposes. Ellipses indicate omitted descriptors and show that only representative fragments of the complete descriptor sets are displayed.

PoSMNA (Pairs of Substances Multilevel Neighborhoods of Atoms) descriptors [29,30], an extension of this approach, represent a pair of substances as a set of paired first- and second-level MNA descriptors derived from the two substances and corresponding to the first and second topological levels of the atomic environment of each central atom (Figure 4). This descriptor type was specifically designed to describe pairs of interacting drugs and is neither a pretrained representation of a drug nor a fusion of representations of the individual molecules within a pair.

Thus, PoSMNA descriptors define pair-specific structural features directly from the structural formulas of two compounds, rather than using pretrained molecular embeddings or combining independently generated representations of individual molecules.

SAR analysis was performed using a special version of PASS DDI (Prediction of Activity Spectra for Substances with respect to Drug–Drug Interactions) [29,30,32]. In PASS DDI, classification models are constructed based on PoSMNA descriptors using an algorithm that is based on a modified naive Bayesian approach [33,34]. After training, PASS DDI calculates probability estimates for various types of biological activity, including ADRs associated with pairs of compounds, based on their structural formulas. No explicit class-rebalancing procedures, including oversampling, undersampling, synthetic-sample generation, class weighting, or cost-sensitive learning, were applied during model development. All PASS DDI models were trained using the original class distributions. The modified naive Bayesian algorithm implemented in PASS has previously been shown to be relatively insensitive to unequal class sizes and incomplete activity annotations [33]. Balanced accuracy was used only as an evaluation metric that assigns equal importance to sensitivity and specificity; it was not used to modify the training samples or to optimize the classification threshold.

In the present work, for a new pair of structures, PASS DDI calculates two probability estimates: *Pa*, which is the probability that a drug pair will cause a specific ADR of a given severity, and *Pi*, which is the probability that the drug pair will not cause an ADR of that severity. The greater the difference *Pa* − *Pi*, the more likely the drug pair is to cause the ADR of the corresponding severity according to the available dataset. PASS DDI does not use a conventional posterior-probability threshold such as *Pa* ≥ 0.5. For each ADR and severity category, the algorithm calculates *Pa* and *Pi* estimates from activity-specific distributions of the internal B-statistic generated using the training data. *Pa* reflects the estimated likelihood that a drug pair belongs to the corresponding activity class, whereas Pi reflects the estimated likelihood of inactivity with respect to that class. Classification was performed using the predefined PASS criterion *Pa* > *Pi*, which was applied uniformly to all ADRs and severity categories. The activity-specific mappings of the B-statistic to *Pa* and *Pi* were derived exclusively from the training data within each cross-validation iteration. Neither test-set labels nor validation metrics were used to select or optimize the decision criterion. Thus, balanced accuracy was not optimized post hoc on the validation data. ROC AUC was additionally reported as a threshold-independent measure of discrimination.

To provide a reference point for the predictive performance of the PASS DDI/PoSMNA approach, several conventional structure-based machine learning methods were additionally evaluated. Because an exhaustive benchmark involving all 14 ADR-specific datasets, multiple severity categories, molecular representations, algorithms, and validation schemes would require substantial computational resources, the comparative analysis was restricted to the bleeding dataset. Bleeding was selected as a representative endpoint because the corresponding dataset contained all four severity categories—Major, Moderate, Minor, and None—and included sufficient numbers of examples for model development and evaluation.

The evaluated methods included a prevalence-based dummy classifier, logistic regression, random forest, and XGBoost. Individual compounds were represented using Morgan fingerprints with a radius of 2 and RDKit topological fingerprints. For each drug pair, the fingerprints of the two compounds were concatenated in a fixed canonical order. Separate binary one-vs-rest models were trained for each bleeding severity category. RDKit version 2025.09.6 was used for molecular fingerprint generation, XGBoost version 3.3.0 was used for gradient-boosted tree models, and scikit-learn version 1.8.0 was used for logistic regression and random forest models.

Class imbalance within the benchmark dataset was handled using balanced class weights for logistic regression and random forest. For XGBoost, the ratio between negative and positive training examples was used to define the scale_pos_weight parameter. No class weighting was applied to the dummy classifier.

The machine learning models were evaluated using two five-fold cross-validation schemes. In drug-disjoint k-fold cross-validation, no individual drug occurring in a test fold was allowed to occur in the corresponding training fold as part of another pair. In conventional pair-wise-stratified k-fold cross-validation, drug pairs were assigned to folds while approximately preserving class proportions, without excluding overlap of individual drugs between the training and test sets. Identical folds were used for all molecular representations and machine learning algorithms within each validation scheme. No decision threshold was optimized using test-set labels. Predictive performance was assessed primarily using ROC AUC.

When constructing the training and testing datasets, we accounted for the specific structure of the data: it consists of not individual compounds, but compound pairs. Therefore, the most widely used cross-validation methods, such as k-fold splitting or leave-one-drug-pair-out validation, could result in information leakage between the training and testing sets and, consequently, unjustifiably overestimated predictive accuracy. Using such an inappropriate validation scheme, a model may rely on information about individual drugs that are frequently associated with a specific effect, rather than learning SAR patterns specific to drug pairs.

**Figure 5:**
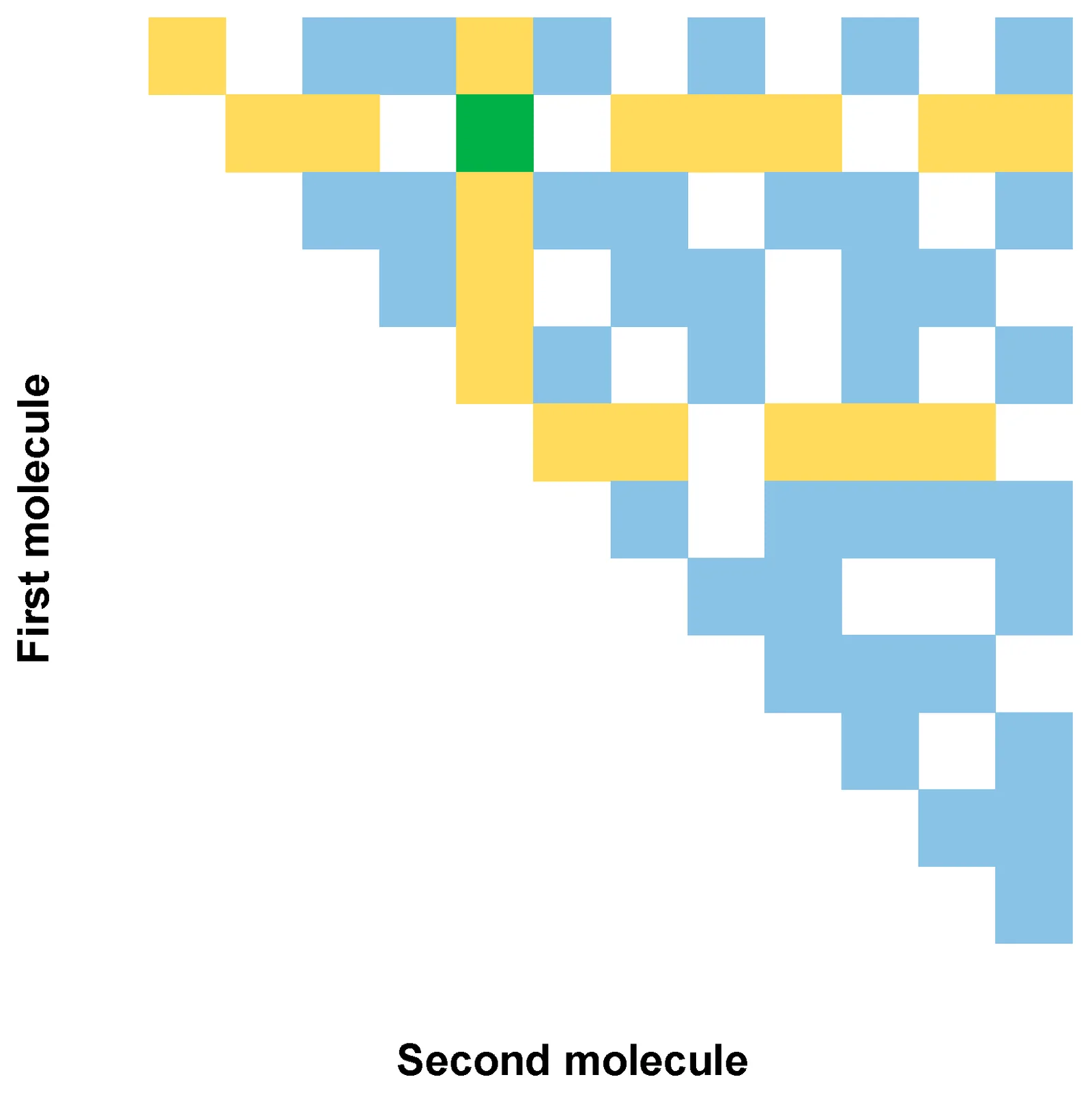
Schematic representation of the “compounds-out” cross-validation procedure. Rows and columns represent individual compounds, whereas cells represent drug pairs. Blue cells indicate pairs included in the training set, green indicates the test pair, yellow indicates pairs excluded from the current validation fold, and white indicates absent data. The procedure prevents any compound occurring in the test pair from appearing in the training set as part of another drug pair.

We previously proposed a modification of the cross-validation procedure that accounts for this dataset structure. We used a splitting procedure that excludes compound-level overlap between the training and testing sets. The cross-validation algorithm is illustrated in Figure 5 and is described in more detail in our previous paper [27]. In this approach, no individual compound present in the test set is included in the training set as part of another pair in each validation fold.

Predictive performance was evaluated using AUC, sensitivity, specificity, and balanced accuracy:

- AUC was calculated as the area under the receiver operating characteristic curve (ROC AUC). It estimates the probability of correctly distinguishing a randomly selected positive example from a randomly selected negative example in the test set, and reflects the model’s ability to discriminate between classes.
- TP (true positives) is the number of correctly classified positive examples.
- TN (true negatives) is the number of correctly classified negative examples.
- FP (false positives) is the number of negative examples incorrectly classified as positive.
- FN (false negatives) is the number of positive examples incorrectly classified as negative.
- Sensitivity is the proportion of correctly classified positive examples among all positive examples, and it is calculated as TP/(TP + FN).
- Specificity is the proportion of correctly classified negative examples among all negative examples, and it is calculated as TN/(TN + FP).
- Balanced accuracy (BA) is the mean of sensitivity and specificity, and it is calculated as (Sensitivity + Specificity)/2.

ADR severity classes were defined as follows:

- Major: severe ADRs that may be life-threatening or require medical intervention.
- Moderate: clinically significant reactions of intermediate severity that may require correction of therapy.
- Minor: mild reactions that generally do not require substantial medical intervention.
- None: no recorded association between the drug pair and the corresponding ADR. This class was treated as conditionally negative and was formed based on the absence of a statistically significant association in the analysis of spontaneous reports.

## 5. Conclusions

This study proposes an approach for predicting the severity of DDI-associated ADRs based on SAR analysis of drug pairs. Using the DrugMAP data, datasets were generated for a number of clinically significant ADRs, including cardiovascular, hepatotoxic, nephrotoxic, hemorrhagic, and neurological effects. We then developed models to predict three severity categories: Major, Moderate, and Minor, together with the conditionally negative None class.

The obtained results show that MNA/PoSMNA molecular descriptors are able to capture relationships between the chemical structures of drug pairs and the severity of associated ADRs. Model performance differed substantially between types of adverse reactions: higher predictive accuracy was achieved for several ADR types that may be associated with a relatively limited range of pharmacological mechanisms, whereas accuracy was lower for complex multifactorial reactions such as hepatotoxicity. This indicates that structural information can be used for a preliminary risk assessment of certain types of ADRs, but could be insufficient for ADRs whose development substantially depends on metabolism, dosing regimens, individual patient characteristics, and the biological context.

Case-based assessment using known clinical cases has shown that the models reproduce the expected risk profiles, including the prediction of an increased probability for relevant ADRs and the assignment of the control combination without a recorded clinically significant interaction to a conditionally negative class. At the same time, the results of this testing emphasize that predicted ADR severity is not always identical to the severity of the outcome in an individual patient. This is because the clinical manifestation of ADRs depends on numerous other factors in addition to the drug itself.

Thus, the developed models can be used as a preliminary screening tool to identify potentially dangerous drug combinations and prioritize cases for further analysis. All models have been implemented in the AdverDDIPred web application, which is part of the Way2Drug platform. This enables their practical use by researchers and specialists in pharmacology, toxicology, medicinal chemistry, pharmacovigilance, and drug safety assessment.

## Figures and Tables

**Table 1:** Predictive performance of PASS DDI models for severity classification of adverse drug reactions associated with drug–drug interactions.

ADR	Class	N	AUC	CI	Sen.	Spec.	BA	AUC_5-Fold_
Cardiac depression	Major	36	0.973	(0.9564–0.9824)	0.917	0.915	0.916	1.000
	Moderate	154	0.996	(0.9933–0.9994)	0.987	0.987	0.987	0.997
	None	61,897	0.982	(0.9733–0.9869)	0.936	0.937	0.937	0.996
CNS depression	None	50,271	0.867	(0.8634–0.8735)	0.786	0.786	0.786	0.987
	Major	1828	0.928	(0.9185–0.9357)	0.877	0.877	0.877	0.995
	Moderate	2873	0.797	(0.7872–0.8065)	0.718	0.718	0.718	0.984
Hyperglycemia	None	45,869	0.936	(0.9035–0.9644)	0.900	0.907	0.905	1.000
	Moderate	103	0.922	(0.8641–0.9467)	0.890	0.894	0.894	1.000
Bradycardia	None	30,102	0.860	(0.8318–0.8788)	0.795	0.795	0.795	0.997
	Major	39	0.620	(0.5555–0.6882)	0.615	0.617	0.616	0.950
	Moderate	89	0.878	(0.8284–0.9397)	0.809	0.813	0.811	0.993
Bleeding	None	39,742	0.895	(0.8911–0.9002)	0.813	0.813	0.813	0.995
	Moderate	1686	0.902	(0.8949–0.9094)	0.817	0.817	0.817	0.993
	Major	816	0.809	(0.8034–0.8220)	0.741	0.742	0.742	0.996
	Minor	54	0.845	(0.7990–0.8881)	0.778	0.769	0.773	0.997
Hypertensive effect	None	34,668	0.918	(0.9062–0.9321)	0.841	0.842	0.842	0.997
	Moderate	287	0.929	(0.9095–0.9539)	0.850	0.849	0.850	0.996
	Major	199	0.874	(0.8517–0.9138)	0.784	0.785	0.784	0.995
Hypotensive effect	None	38,162	0.911	(0.9069–0.9156)	0.833	0.833	0.833	0.994
	Moderate	3459	0.919	(0.9113–0.9235)	0.840	0.840	0.840	0.997
	Major	44	0.894	(0.8440–0.9422)	0.841	0.840	0.841	0.974
Hepatotoxicity	None	52,593	0.794	(0.7869–0.8021)	0.730	0.730	0.730	0.994
	Moderate	1915	0.730	(0.7208–0.7364)	0.678	0.678	0.678	0.994
	Major	900	0.770	(0.7485–0.7866)	0.708	0.708	0.708	0.999
QT-interval prolongation	None	34,081	0.860	(0.8557–0.8633)	0.791	0.791	0.791	0.989
	Major	6286	0.849	(0.8442–0.8538)	0.770	0.770	0.770	0.984
	Moderate	6082	0.790	(0.7849–0.7965)	0.726	0.726	0.726	0.977
Nephrotoxicity	None	75,900	0.930	(0.9247–0.9412)	0.880	0.876	0.876	1.000
	Major	423	0.900	(0.8874–0.9135)	0.827	0.828	0.828	0.996
	Moderate	811	0.928	(0.9154–0.9393)	0.869	0.869	0.869	0.995
Immunosuppressive effect	None	96,095	0.790	(0.7835–0.8058)	0.722	0.721	0.722	1.000
	Major	504	0.761	(0.7420–0.7766)	0.698	0.698	0.698	0.998
	Moderate	372	0.712	(0.6734–0.7475)	0.669	0.670	0.670	0.998
Myelosuppressive effect	None	76,164	0.920	(0.8965–0.9333)	0.850	0.849	0.850	0.996
	Major	165	0.682	(0.6024–0.7488)	0.691	0.688	0.690	0.975
	Moderate	298	0.944	(0.9331–0.9585)	0.879	0.880	0.879	0.996
Hyperkalemia	None	90,942	0.890	(0.8602–0.9206)	0.820	0.825	0.824	0.990
	Moderate	124	0.853	(0.8161–0.8893)	0.766	0.770	0.768	0.991
	Major	141	0.897	(0.8618–0.9292)	0.844	0.845	0.844	0.999
Hypokalemia	None	61,972	0.950	(0.9280–0.9907)	0.909	0.907	0.908	0.982
	Minor	120	0.977	(0.9670–0.9875)	0.942	0.938	0.940	1.000
	Moderate	15	0.921	(0.8284–0.9976)	0.933	0.932	0.932	0.927

ADR: adverse drug reaction; Class: DDI severity class; N: number of drug pairs belonging to the corresponding class in the dataset; AUC: area under the receiver operating characteristic curve obtained using compounds-out cross-validation; CI: 95% confidence interval for AUC; Sen.: sensitivity; Spec.: specificity; BA: balanced accuracy; AUC_5-fold_: area under the receiver operating characteristic curve obtained using conventional pair-wise-stratified five-fold cross-validation. Classification was performed using the predefined PASS criterion *Pa* > *Pi*.

**Table 2:** Unweighted macro-average predictive performance of ADR-specific models by severity class.

Class	No. of ADR Models	Mean N per Model	AUC	Sen.	Spec.	BA
		Mean by Class				
Major	12	948	0.83	0.776	0.776	0.776
Moderate	2	87	0.911	0.86	0.853	0.857
Minor	14	1305	0.873	0.817	0.817	0.817
None	14	56,318	0.893	0.829	0.829	0.829

Class: DDI severity class; No. of ADR models: number of ADR-specific models contributing to the corresponding severity-class average; Mean N per model: mean number of drug pairs belonging to the corresponding severity class across the contributing ADR-specific models; AUC: area under the receiver operating characteristic curve; Sen.: sensitivity; Spec.: specificity; BA: balanced accuracy.

**Table 3:** Comparison of PASS DDI/PoSMNA and conventional structure-based machine learning methods used in the bleeding benchmark.

Method	Input Data	Molecular Representation	Classification Approach	Class Imbalance Handling	Interpretability	Relative Computational Complexity
PASS DDI	2D structures of both drugs	PoSMNA	Modified naive Bayesian approach	No explicit resampling or class weighting	*Pa/Pi* estimates; pair-specific structural descriptors	Moderate
Dummy	Class labels	None	Prevalence-based prediction	None	High	Very low
Logistic regression	2D structures of both drugs	Morgan/RDKit fingerprints	Linear binary classifier	Balanced class weights	Moderate	Low
Random forest	2D structures of both drugs	Morgan/RDKit fingerprints	Tree ensemble	Balanced class weights	Moderate	Moderate
XGBoost	2D structures of both drugs	Morgan/RDKit fingerprints	Gradient-boosted trees	scale_pos_weight	Moderate	Moderate–high

Note: PoSMNA, Pairs of Substances Multilevel Neighborhoods of Atoms. Relative computational complexity is reported qualitatively and refers to the implementations evaluated in the present benchmark.

**Table 4:** Case-based assessment of predicted DDI-associated ADR severity using clinically documented drug combinations. *Pa* and *Pi* denote the PASS probability estimates for activity and inactivity, respectively. A severity category is considered predicted when *Pa* > *Pi*. The presented cases are illustrative and do not constitute an independent external validation dataset.

Drug 1	Drug 2	Known Effect	Major *Pa/Pi*	Minor *Pa/Pi*	Moderate *Pa/Pi*	None *Pa/Pi*	Prediction Assessment
Warfarin	Amiodarone	Bleeding	0.090/0.691	0.803/0.075	0.767/0.023	0.050/0.600	Severity underestimated (Major false negative)
Warfarin	Aspirin	Bleeding	0.942/0.035	0.837/0.061	0.729/0.030	0.006/0.965	Consistent with clinical evidence
Azithromycin	Diltiazem	QT-interval prolongation on ECG	0.127/0.337	-	0.665/0.072	0.130/0.342	Association detected; severity potentially underestimated
Quetiapine	Escitalopram	QT-interval prolongation on ECG	0.932/0.007	-	0.631/0.085	0.008/0.886	Consistent with clinical evidence
Levothyroxine	Amlodipine	Combination without a reported clinically significant DDI	0.003/0.853	-	0.021/0.902	0.876/0.012	Consistent with absence of reported clinically significant DDI
Tramadol	Venlafaxine	Serotonin syndrome; CNS toxicity	0.237/0.052	-	0.501/0.119	0.076/0.400	Consistent with clinical evidence

## Data Availability

The source data used in this study are available from the DrugMAP and TwoSides resources. The developed prediction models are available through the Way2Drug resource at https://way2drug.com/AdverDDIPred. The processed datasets generated during the current study are available from the corresponding author upon request.

## Abbreviations

The following abbreviations are used in this manuscript:

ADR	adverse drug reaction
AUC	area under the receiver operating characteristic curve
BA	balanced accuracy
CNS	central nervous system
DDI	drug–drug interaction
ECG	electrocardiogram
FAERS	FDA Adverse Event Reporting System
FDA	U.S. Food and Drug Administration
FN	false negative
FP	false positive
N	Number of drug pairs belonging to the class in the dataset
MNA	multilevel neighborhoods of atoms
*Pa*	probability “to be active”
*Pi*	probability “to be inactive”
PASS	Prediction of Activity Spectra for Substances
PoSMNA	Pairs of Substances Multilevel Neighborhoods of Atoms
QTc	corrected QT interval
ROC	receiver operating characteristic
SAR	structure–activity relationship
Sen.	sensitivity
Spec.	specificity
TN	true negative
TP	true positive
